# Remote Patient Monitoring and Teleconsultation to Improve Health Outcomes and Reduce Health Care Utilization of Pediatric Asthma (ALPACA Study): Protocol for a Randomized Controlled Effectiveness Trial

**DOI:** 10.2196/45585

**Published:** 2023-07-03

**Authors:** Mattienne van der Kamp, Vera Hengeveld, Nico Willard, Boony Thio, Pascal de Graaf, Inge Geven, Monique Tabak

**Affiliations:** 1 Pediatric Department Medisch Spectrum Twente Enschede Netherlands; 2 Biomedical Signals and Systems Department University of Twente Enschede Netherlands; 3 Remote Patient Management and Chronic Care Department Philips Research Eindhoven Netherlands

**Keywords:** asthma, children, telemedicine, home monitoring, randomized controlled trial, health care utilization, health care costs, protocol, spirometry, adherence, nebulizer, pediatric care, utilization, remote monitoring, asthma care

## Abstract

**Background:**

Childhood asthma is imposing a great financial burden on the pediatric health care system. Asthma costs are directly related to the level of asthma control. A substantial part of these costs may be preventable by the timely and adequate assessment of asthma deterioration in daily life and proper asthma management. The use of eHealth technology may assist such timely and targeted medical anticipation.

**Objective:**

This paper describes the Ambulatory Pediatric Asthma Care (ALPACA) study protocol to investigate the effectiveness of an eHealth intervention consisting of remote patient monitoring and teleconsultation integrated into the daily clinical care of pediatric patients with asthma. This intervention aims to reduce health care utilization and costs and improve health outcomes compared to a control group that receives standard care. In addition, this study aims to improve future eHealth pediatric asthma care by gaining insights from home-monitoring data.

**Methods:**

This study is a prospective randomized controlled effectiveness trial. A total of 40 participants will be randomized to either 3 months of eHealth care (intervention group) or standard care (control group). The eHealth intervention consists of remote patient monitoring (spirometry, pulse oximetry, electronic medication adherence tracking, and asthma control questionnaire) and web-based teleconsultation (video sharing, messages). All participants will have a 3-month follow-up with standard care to evaluate whether the possible effects of eHealth care are longer lasting. During the entire study and follow-up period, all participants will use blinded observational home monitoring (sleep, cough/wheeze sounds, air quality in bedroom) as well.

**Results:**

This study was approved by the Medical Research Ethics Committees United. Enrollment began in February 2023, and the results of this study are expected to be submitted for publication in July 2024.

**Conclusions:**

This study will contribute to the existing knowledge on the effectiveness of eHealth interventions that combine remote patient monitoring and teleconsultation for health care utilization, costs, and health outcomes. Furthermore, the observational home-monitoring data can contribute to improved identification of early signs of asthma deterioration in pediatric patients. Researchers and technology developers could use this study to guide and improve eHealth development, while health care professionals, health care institutions, and policy makers may employ our results to make informed decisions to steer toward high-quality, efficient pediatric asthma care.

**Trial Registration:**

ClinicalTrials.gov NCT05517096; https://clinicaltrials.gov/ct2/show/NCT05517096

**International Registered Report Identifier (IRRID):**

PRR1-10.2196/45585

## Introduction

### Background

Asthma is one of the most common pediatric chronic diseases with a prevalence between 7% and 10% [1,2]. Childhood asthma imposes a significant physical and social burden on the affected child and their family. It may prevent children from participating in play and recreational activities, hamper social contacts, and lead to school absence and reduced school performance [2-4]. As a result, children with asthma have a reduced quality of life (QoL) in comparison to their healthy peers [5-7].

Asthma is also imposing a great financial burden on the pediatric health care system [8]. High-income countries spend between 1% and 2% of their health care budget on asthma [1,9]. For example, in the Netherlands, direct pediatric asthma health care costs have doubled over the last 2 decades and amounted to €90.8 million (US $92 million) in 2017 [10], compared to US $5.92 billion in the United States in 2013 [11]. Hospital and pharmacy costs occupy an increasing share and are the primary contributors to the total health care costs [10,11]. Additionally, pediatric asthma also exerts a large indirect economic burden due to missed school and workdays [12]. The economic burden of asthma is disproportional along with the severity of the disease [13]. Moreover, asthma costs are directly related to the level of asthma control, as poor control is associated with more hospitalizations, emergency visits, and other health care contacts [14].

A substantial part of these costs may be preventable and asthma outcomes can be improved if we are able to overcome existing challenges in pediatric asthma care. These challenges include (1) a limited opportunity for health care professionals (HCPs) to retrieve objective insight into the severity and dynamics of asthma symptoms in daily life [15], (2) the difficulty of attuning an optimal treatment that not only treats the intermittent symptoms but also the underlying inflammation sufficiently [16], (3) low adherence and poor inhaler technique of asthma therapy in children with asthma [17,18], and (4) inadequate assessment of severity or failure of the family to call for help when required [19].

The use of eHealth technology, like remote patient monitoring and teleconsultation, may help overcome these challenges [20]. In recent years, the development and validation of these types of eHealth technologies for pediatric asthma have skyrocketed [21-23]. Studies cautiously show evidence that eHealth can improve health outcomes and QoL and that it can be cost-effective [22,24,25]. However, the generalizability of these results is hindered by high heterogeneity in study end points and designs and poor quality of evidence [26-28]. Moreover, these results are often limited to a single disease management or monitoring domain (eg, medication adherence [29]), but asthma is a multifaceted disease with variable manifestations influenced by asthma severity, the level of adequate disease management, and the influence of (environmental) triggers [2,30]. There is limited scientific evidence that provides insights into the combination of monitoring parameters and interventional tools to predict early signs of asthma deterioration in a timely manner [31,32]. A recent study showed that remote patient monitoring may identify cues and causes of asthma control deterioration at home and help provide insights into patterns of asthma [33]. Furthermore, another study showed that teleconsultation and remote patient monitoring allows patients and caregivers to rapidly regain asthma control through timely and targeted medical anticipation, thereby progressing from reactive to proactive therapy and preventing asthma exacerbation [34].

The research gap, therefore, lies in the evaluation of pediatric asthma eHealth strategies consisting of a multidomain remote patient monitoring and teleconsultation approach while using a high-quality research design with relevant clinical end points on health care utilization, asthma outcomes, and QoL [26].

### Objectives

In this study, called Ambulatory Pediatric Asthma Care (ALPACA), we will investigate an eHealth intervention for pediatric asthma care, in which multidomain, objective remote patient monitoring (ie, lung function, therapy adherence, and oxygen saturation) and teleconsultation (ie, web-based app to chat with HCPs, symptom questionnaires, and share videos) are integrated into clinical care and the patients’ daily lives for 3 months.

The main study objective is to investigate whether children with asthma who use this eHealth intervention have reduced asthma health care utilization and costs compared to a control group that receives standard care for 3 months.

We will investigate the effects of this eHealth intervention on asthma outcomes (ie, asthma control, lung function, and the number of exacerbations), therapy adherence, self-management, and QoL after the intervention period of 3 months and at the 3-month follow-up. Overall use, acceptance, and satisfaction will be evaluated as well.

To enable further improvement of pediatric eHealth asthma care, we will exploratively investigate possible relations between objective home-monitoring parameters and asthma outcomes, the relation between self-reported dyspnea and home-measured lung function, and possible early signals in the preluding period before an asthma exacerbation. Moreover, we will explore the contribution of different eHealth intervention elements to asthma outcomes, patient-reported outcomes, and decision-making and identify patient characteristics of participants with successful eHealth care outcomes, such as health care utilization and adherence.

### Hypothesis

Based on the recent literature [22] and results from previous studies [33-35], we hypothesize that over 3 months, compared to standard care, the ALPACA eHealth intervention will lead to a significant reduction in health care utilization and costs with at least equal asthma outcomes and QoL.

## Methods

### Study Design

The ALPACA study is a prospective 2-arm randomized controlled effectiveness trial of an eHealth intervention in pediatric patients with asthma, which builds on a previous eHealth feasibility trial [34]. Participants will be randomized to either eHealth care (ie, intervention group) or standard care (ie, control group). During the 3-month follow-up, all patients will receive standard care and blinded observational home monitoring to evaluate the sustained effects of eHealth compared to the control group. The study protocol was designed in accordance with the SPIRIT (Standard Protocol Items: Recommendations for Interventional Trials) guidelines (Table 1) [36].

**Table 1:** Schematic SPIRIT (Standard Protocol Items: Recommendations for Interventional Trials) schedule of enrollment, interventions, and assessments.

Time point		Study period					
		Enrollment	Allocation/first visit	Postallocation			Closeout/end visit
		−t_1_	t_0_	t_1_ Intervention period: 3 months	t_2_ middle visit	t_3_ follow-up period: 3 months	t_e_
**Enrollment**							
	Eligibility screen	✓					
	Informed consent	✓					
	Instruction and installment eHealth		✓				
	Allocation		✓				
**Interventions**							
	eHealth care (the Engage web portal, spirometry, pulse oximetry, smart inhaler)			✓(A^a^)			
	Standard care + observational monitoring (spirometry, pulse oximetry, smart inhaler)			✓(B^a^)		✓	
	Observational monitoring (cough sensor, air sensor, sleep monitor)			✓		✓	
**Assessments**							
	Baseline variables (age, gender, BMI, *z* score, GINA^b^ medication step, allergic rhinitis, asthma-related medical history, and baseline spirometry, such as FEV1^c^ and FVC^d^	✓	✓				
	Medical utilization data Number of visits (planned/ emergency/hospital admissions), diagnostic tests, telephonic/internet consultations, total health care costs			✓		✓	
	Questionnaire evaluations: health literacy (HLS^e^), asthma control (C-ACT^f^), quality of life (PAQLQ^g^ and EQ-5D-Y), self-management (PAM-13^h^)		✓		✓		✓
	Quality of care (CSQ^i^)				✓		✓
	Monitoring data: medication use, sleep, nocturnal heart/respiratory rate, nocturnal coughing/ wheezing, air quality, lung function, and oxygen saturation			✓		✓	
	Intervention data: intervention use logs, and nebulizer use			✓(A^a^)			
	eHealth–specific evaluations: system usability (SUS^j^), technology acceptance (TAM^k^), and interview				✓(A^a^)		

^a^Depending on the randomization. Group A receives eHealth care, and group B receives standard care within the intervention period (t_1_).

^b^GINA: Global Initiative for Asthma.

^c^FEV1: forced expiratory volume.

^d^FVC: forced vital capacity.

^e^HLS: Health Literacy Survey.

^f^C-ACT: Childhood Asthma Control Test.

^g^PAQLQ: Pediatric Asthma Quality of Life Questionnaire.

^h^PAM-13: 13- Item Patient Activation Measure.

^i^CSQ: Client Satisfaction Questionnaire.

^j^SUS: System Usability Scale.

^k^TAM: Technology Acceptance Model.

### Ethics Approval

This study protocol was approved by the Medical Research Ethics Committees United (NL74559.100.21) and registered in ClinicalTrials.gov (NCT05517096). The data handling and storage will comply with the European Union (EU) General Data Protection Regulation (GDPR).

### Study Setting and Eligibility Criteria

A total of 40 children with moderate-to-severe pediatrician-diagnosed asthma between 4 and 11 years of age will be recruited from the Medisch Spectrum Twente (MST) hospital in Enschede, the Netherlands. Pediatrician-diagnosed asthma includes confirmation of asthma diagnosis with a positive reversibility or challenge test [2]. Children are classified as having moderate or severe asthma if they meet one of the criteria shown in Table 2. Children with comorbid chronic diseases, previous asthma eHealth care intervention trial experiences [34], or children/parents with an inability to understand or speak Dutch are not eligible to participate. Additionally, children that do not live for more than 80% at the same address or those who do not have a stable Wi-Fi connection at home are excluded from participation.

Participants will be recruited from the pediatric department of MST using consecutive sampling, which is expected to take 40 weeks. A study flyer will be displayed in the pediatric ward and distributed among pediatric patients with asthma after an outpatient visit. Patients who indicate that they may be contacted will be contacted after 1 week, but patients may also contact the researchers themselves for further verbal and written study information. Offline written informed consent from parents will be obtained prior to participation in this study.

**Table 2:** Criteria for classification as moderate or severe asthma.

Condition	Moderate asthma	Severe asthma
Medication	GINA^a^ step 3/4 or uncontrolled (C-ACT^b^<20) in GINA step 2 [2]	Step 5 or uncontrolled (C-ACT<20) despite medium to high-dose ICS^c^-LABA^d^ in step 4
Recent exacerbation (<3 months) despite controller medication	Needing a salbutamol regimen of oral cortical steroid therapy	Leading to a hospital admission
Bronchial hyperreactivity assessed with an exercise challenge test in cold air [37,38]	Between 25% and 50% decrease in FEV1^e^ [39]	>50% decrease in FEV1 [39]

^a^GINA: Global Initiative for Asthma.

^b^C-ACT: Childhood Asthma Control Test.

^c^ICS: inhaled corticosteroid.

^d^LABA: long-acting beta2-agonist.

^c^FEV1: forced expiratory volume.

### Randomization and Blinding

Patients will be randomized to either eHealth care (intervention group) or standard care (control group) during the intervention period (t_1_) by independent block randomization at the first home visit. The electronic data capture (EDC) software Castor (Castor) will allocate the participants using stratified block randomization with multiple block sizes (2, 4, and 6) into either group A or group B with an allocation ratio of 1:1 [40]. Castor randomly mixes these block sizes and hides the block size from the executor, which guarantees concealed allocation [40]. Randomization will be stratified on the number of hospital visits concerning asthma in the past 6 months (using 4 strata: 0-1 visit, 2-3 visits, 4-5 visits, or 6 or more visits), as it is known that previous exacerbations and health care utilization are risk factors for future exacerbations [2,41]. From this point, the blinding will be broken as the researchers, HCPs, and patients will need to know whether they should prepare for, provide, or receive eHealth care, respectively.

### Intervention

The eHealth care intervention, consisting of remote patient monitoring and teleconsultation, was designed to detect loss of asthma control timely and accurately in daily life, increase awareness about the severity of asthma symptoms, and improve the safety of care for both physicians and patients by using objective measurements as the basis for shared decision-making. The technologies that are used for this eHealth care are a web-based patient portal, remote monitoring devices, and home-nebulizer therapy. This intervention was designed based on previous work on multidomain monitoring [33] and clinically implemented eHealth care [34].

### eHealth Technologies

#### Web-Based Patient Platform

For the eHealth care, component a digital platform called Engage will be used that allows HCPs and patients to collaborate by facilitating web-based communication, displaying home measurements and questionnaire data, and allowing HCPs to share educational content. The configuration of the patient platform was specifically developed for this study in collaboration with the HCPs using the elements of the customizable Philips VitalHealth Engage platform (Royal Philips). This is a certified platform according to the European Medical Device Regulation; it is Conformité Européenne (CE) certified, classified as a class IIa medical device, and has been used in several clinical trials.

For patients, the Engage platform involves a personal page designed to provide children/parents the opportunity to unobtrusively share a request for help and integrate time- and event-based repeated measures or diary entries [42]. The page includes a chat function to allow the children/parents and HCPs to communicate in real time or according to their availability and wishes. The participants also have the option to share photos, sound recordings, or video files with the HCP (eg, to aid in the clarification of symptoms). Participants can enter their spirometry and oxygen saturation (SpO_2_) measurements into the platform. Additionally, participants will be requested to fill in the Childhood Asthma Control (C-ACT) questionnaire on their page weekly.

**Figure 1:**
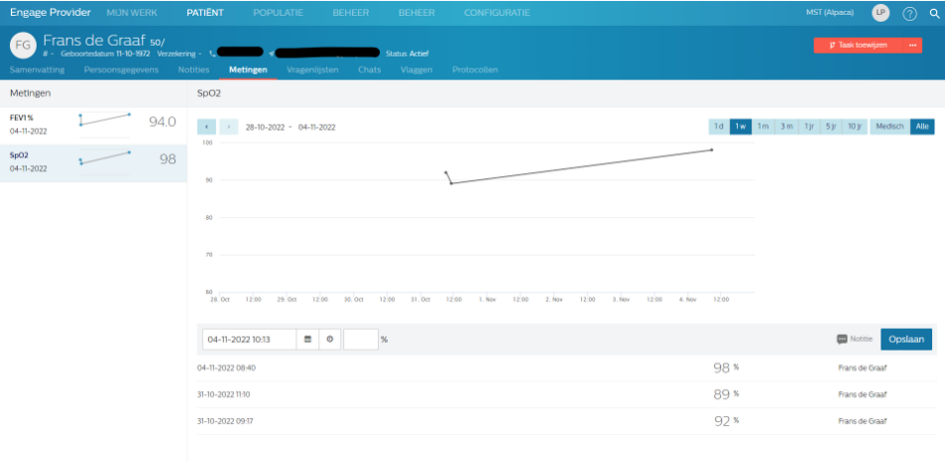
Example of health care professional (HCP) dashboard for lung function and oxygen saturation measurement of a specific patient.

For HCPs, the platform provides access to a dashboard for each patient, which displays trends of the measurements and allows access to details of measurements and questionnaires (Figure 1).

#### Remote Monitoring Devices

**Figure 2:**
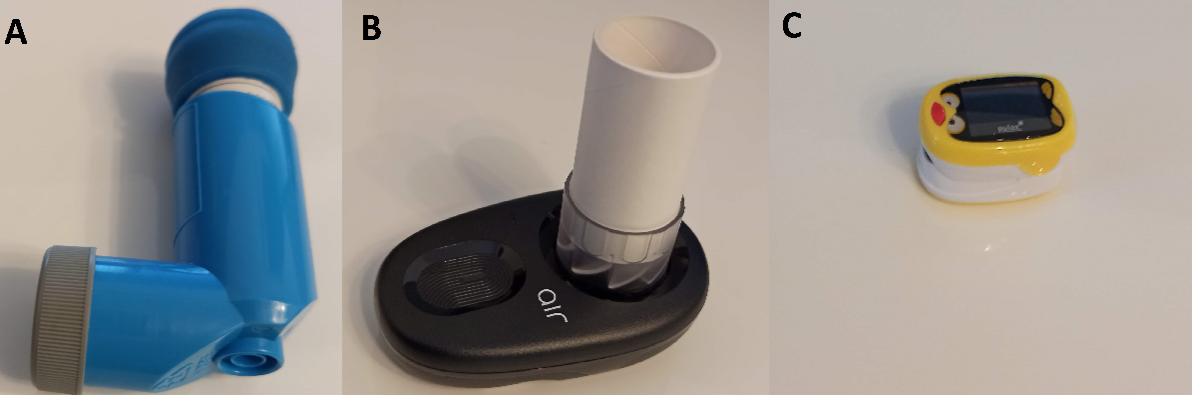
Interventional monitoring devices: from left to right: A) FindAir ONE smart inhaler to track rescue use and controller adherence. B) NuvoAir Air Next spirometer to track lung function. C) Pulox PO-210B pulse oximeter to monitor oxygen saturation during exacerbation.

Our study combines several remote monitoring devices to actively monitor the participants in the intervention group during the eHealth care component. A total of 3 interventional monitoring devices, FindAir ONE smart inhaler (FindAir Sp), NuvoAir Air Next spirometer (NuvoAir AB), and Pulox PO-210B pulse oximeter (Novidion Gmbh; Figure 2), as well as a smartphone, will be provided to the children and their parents. Data reports will be shared by the participant through the Engage platform and automatically stored and accessible for HCPs through web-based cloud storage.

The FindAir ONE is a smart inhaler cap that will be placed on top of metered dose inhalers of the participants. It will always be used in combination with valved holding chambers. The sensor continuously collects data on the date and timestamps of inhalation, which is automatically synced and can be assessed in real time by both the patient and HCP on the FindAir platform.

NuvoAir Air Next is a handheld spirometer designed for home monitoring of lung function, with a built-in temperature sensor to compensate for the flow and volume for the body temperature pressure saturated correction [43]. This measurement will be performed twice per week or upon indication of the HCPs or the participants themselves during symptoms.

The Pulox PO-210B is a children’s edition of SpO_2_ pulse oximeter. It will be used offline as needed within the intended use for clinical reasons, in case of asthma exacerbations to monitor SpO_2_, and if the lung function measurement quality is poor.

**Figure 3:**
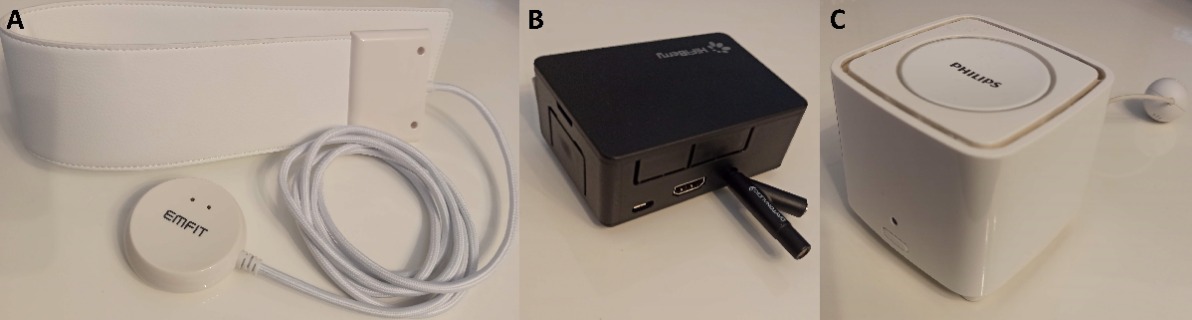
Investigational observational monitoring devices: from left to right: A) Emfit QS+ACTIVE ballistography sleep sensor to track sleep. B) Philips cough and wheeze monitor to track respiratory sounds during sleep. C) Philips Snifferbee air sensor to track air quality in participants’ bedrooms.

In addition, and not part of the intervention, 3 investigational devices, namely, an Emfit QS+ACTIVE sleep monitor (Emfit Ltd), a Philips cough monitor, and a Philips Snifferbee air sensor (Figure 3), will exclusively be used for observational research purposes in both the intervention and standard care group and follow-up of this study. The observational data of the investigational devices will therefore be stored in the cloud (Emfit QS platform and Philips platform). The data will not be shown to the HCPs and patients and only be used to investigate possible relations between objective home monitoring parameters and asthma outcomes.

The Emfit QS+ACTIVE is a commercially available contactless sleep monitor that can provide a detailed description of sleep quality using ballistography [9]. The sensor is installed under the mattress to monitor activity, heart rate, and respiration rate at night and has been validated and previously used for sleep studies in both adults and children [44-46]. The device is CE marked and will be used for its intended use.

The Philips cough monitor is a device intended to store and transmit respiratory sound features and snippets (1 second), which are measured by the microphone in the device. The device will be placed next to the bedside to monitor nocturnal coughing and wheezing.

Snifferbee is Philips’ air quality monitor prototype. It consists of multiple sensors to detect air quality and identify triggers for asthmatic reactions. Snifferbee can measure particles in the room air of 2.5 µm (optical scattering sensor), along with temperature, humidity, and carbon dioxide levels (with a nondispersive infrared sensor). The SnifferBee will be used within its intended use to measure air quality in the participants’ bedrooms.

#### Home Nebulizer Therapy

**Figure 4:**
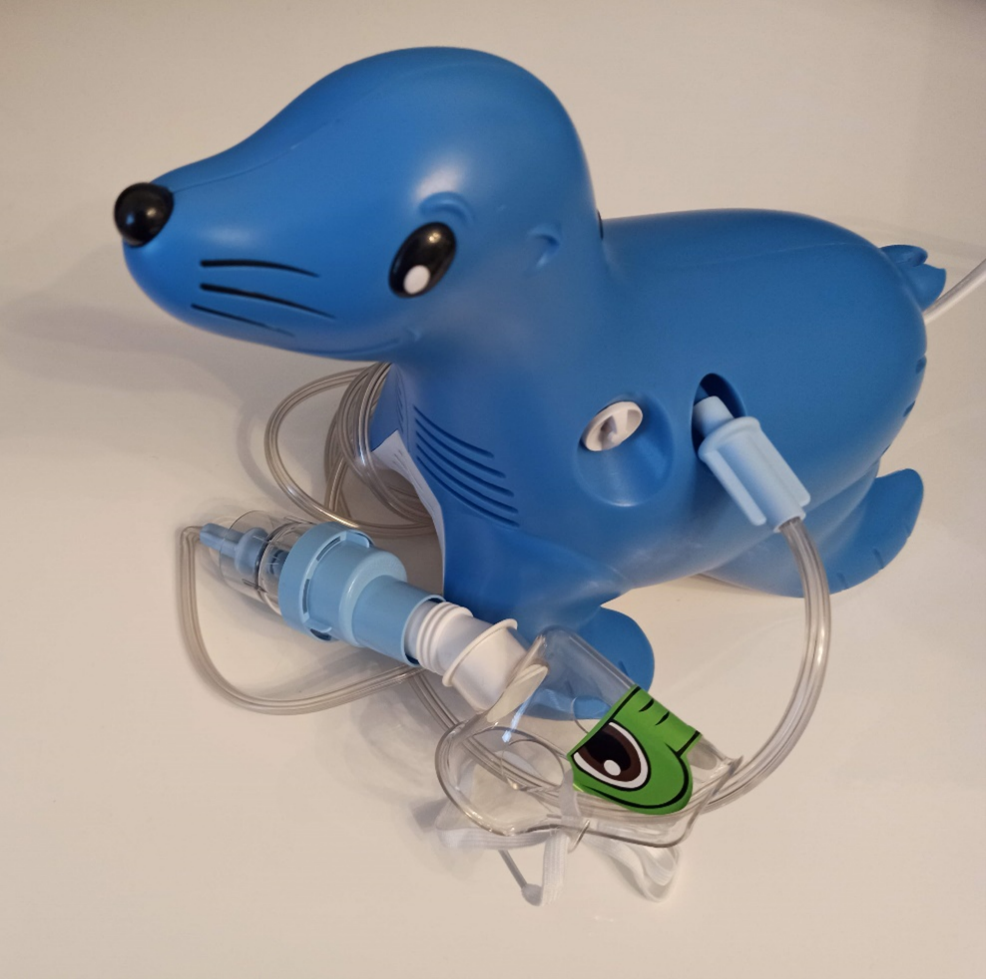
Philips Sami the Seal home nebulizer.

All participants will receive a Philips Sami the Seal nebulizer for the intervention phase and will be trained and supervised for correct use and nebulizer hygiene (Figure 4) [47]. Nebulizing therapy is often started when bronchodilator inhalers do not have the expected effect anymore or in the hospital during an acute exacerbation. During the intervention phase of this study, nebulizing therapy may be started at home under the supervision of an experienced HCP (via video call). Criteria for starting nebulizing therapy under supervision at home during the intervention period are (1) SpO_2_ ≤94% without significant effect of the reliever inhaler medication (as recommended by the Dutch Pediatricians Association) [48]; (2) reproducible lung functions <50% predicted, without significant effect of the reliever inhaler medication (defined as an increase of at least 10% and increase to lung function above 50% predicted); or (3) on the advice of the pulmonary pediatrician.

### eHealth Care Intervention

#### Intervention Group

Participants will be in contact with the HCPs via the web-based Philips Engage platform (Figure 1). HCPs will provide care according to current care standards [2,48] while applying a state-of-the-art web-based care pathway [34]. Within this care pathway, HCPs aim to build up the confidence of the participants and their parents by enhancing their understanding and self-management of the disease via feedback on the web-based communication platform substantiated with monitoring data during real-life events. The key focus will be on important asthma management issues, such as symptom perception, therapy adherence, inhalation techniques, trigger control, and acute asthma management [2]. Participants will be repeatedly and pragmatically supported and coached using elements from behavioral theories (eg, self-monitoring, goal setting, immediate feedback, contingency management) and be involved in shared management to progress on an individual pace toward self-assessment and self-management [49].

During the eHealth intervention, HCPs (ie, nurse practitioners, a technical physician, and a pediatric pulmonologist) will be notified in real time of new communications on the Engage platform by push notifications and will respond at least within 1 workday to allow for timely anticipation. Participants will be instructed to not wait for communications in the case of an emergency and to proceed to emergency care. Moreover, once a week, the HCPs will have a multidisciplinary meeting to discuss data trends and patient communications to adjust the individual treatment strategies as necessary. HCPs will always have the option to invite patients for a hospital visit if this is needed to fulfill the current care standards [2,48].

#### Standard Care Control Group

Participants in the control group will receive standard care and therefore will not receive the home nebulizer. The spirometer, pulse oximeter, and smart inhalers will be provided but only used for observational monitoring in the control group, similar to the investigational devices (cough monitor, air quality sensor, and sleep monitor). The control group will be able to use the Engage portal to share measurements and questionnaire data, but they will not receive any feedback or data interpretation. This same protocol holds for the 3-month follow-up period to be able to assess differences in the primary and secondary outcome parameters after cessation of the intervention.

### Outcome Measures

Demographic characteristics (age, gender, medication use, other medication use, allergy due to aeroallergens, eczema, doing sports, and smoking behavior at home) will be retrieved from electronic patient records.

The primary outcome variables are health care utilization and costs. All health care activities (ie, hospital admissions, outpatient visits, emergency visits, diagnostic activities, etc) will be labeled using the Dutch health care registration system of the Dutch Healthcare Authority. The accompanying health care costs will be calculated using activity-based costing [50]. Furthermore, the specific eHealth care costs will be calculated using bottom-up gross costing, consisting, for example, of the working hours of the HCPs and the depreciation costs of the monitoring devices [50].

Multimedia Appendix 1 contains the description and analysis of other outcome variables to investigate the effects of the eHealth intervention, which will enable research that can further improve pediatric eHealth asthma care.

All data will be handled according to the EU GDPR and the Dutch Act on the Implementation of the GDPR and securely stored in the Castor EDC system.

### Sample Size

Previous data from an eHealth clinical feasibility study (N=30) using a comparable intervention within the same patient population revealed a reduction in health care utilization of 80% [34]. This data showed an average reduction in the number of outpatient consultations from 3.9 to 0.7 (SD 1.8) per patient per half year.

A power simulation was performed for a Mann-Whitney-Wilcoxon 2-sided test. The health care utilization was assumed to be Poisson distributed with means according to the clinical feasibility study. For the control group, the Poisson rate was equated to the historical data of the pilot divided by 2 to account for the study durations. The intervention group’s rate was taken to be the postdata of the feasibility study divided by 2. As the ALPACA study makes use of the intention-to-treat principle, noncompliance to the intervention was included in the simulation. The number of nonadherent participants was random in the simulation according to a binomial distribution (similar to the clinical feasibility study data, with 3 out of 33 participants). This was used to calculate the control group rate, which is a conservative estimate of no benefit from the intervention. Together with the assumption of an Alpha Type 1 error of 0.05, the sample size calculation results in 36 (18 intervention and 18 controls), with a power of 97.1% for the primary outcome and 80% for secondary follow-up effects (assuming a lasting effect of 80%). Accounting for dropouts (n=4), we would need to include 40 children.

### Study Procedures

After enrollment (−t_1_), an HCP will perform a home visit (t_0_) to allocate the participant to the intervention or control group, install the monitoring devices, and carefully instruct participants about the use of the eHealth platform and monitoring devices. All oral instructions will be complemented with written and digital instruction flyers to ensure that all the instructed information can be retrieved afterward. A second home visit (t_2_) is planned after the intervention period to check all devices. Moreover, an in-person semistructured interview will be held to evaluate the user experience of the interventional and investigational technology together with filling in the feasibility and acceptance questionnaires (Multimedia Appendix 1). A third, final home visit (t_e_) will be held at the end of the study after follow-up (t_3_) to retrieve all monitoring devices and evaluate this period via the evaluation questionnaires (Multimedia Appendix 1).

### Data and Statistical Analyses

Descriptive statistics will be used to examine all continuous outcome measures expressed in mean and SD for normally distributed variables and with median and IQR for nonnormal distributed variables. Univariate analyses will be performed with SPSS software (version 22.0; IBM Corp). The homogeneity of variances will be verified with the Levene test. Histogram plots and the Shapiro-Wilk test will be used to determine whether the variables are normally distributed among the groups. A *t* test (or nonparametric Mann-Whitney U test) will be used to compare the primary outcomes between eHealth care and standard care, and *P≤*.05 will be considered significant. The analysis will be performed according to the intention-to-treat principle [51], and missing data will be handled with pairwise deletion. An interim analysis will be conducted after half the participants are done with the intervention. In case the interim analysis reveals a significant increase in health care utilization in the intervention group, this study will be prematurely ended.

## Results

This study was approved by the Medical Research Ethics Committees United on August 11, 2022, and by the institutional review boards of Philips on August 8, 2022, and MST on January 11, 2023.

The expected timeline of the ALPACA study is 15 months, including an enrollment period of 9 months and a study duration of 6 months. As of August 2023, we expect to start enrollment, which will make data collection compete in May 2024. The results are expected shortly afterward in July 2024.

## Discussion

### Expected Knowledge Contribution

Childhood asthma is imposing a great financial burden on the pediatric health care system, but a substantial part of the costs may be preventable by timely and adequate medical anticipation using eHealth. Therefore, In the ALPACA study, we will investigate whether children with asthma who use an eHealth intervention have reduced asthma health care utilization and costs compared to the control group. This study protocol applies a combination of relevant monitoring domains of pediatric asthma based on the recent literature [22,33,34] and guidelines [2]. Primary validated monitoring devices will be combined with web-based communication between HCPs and patients to enable pediatricians to make well-informed and substantiated treatment decisions and allow targeted support for optimal disease management. This randomized controlled effectiveness trial design captures an extensive prospective longitudinal home monitoring data set, which allows the evaluation of the effect of eHealth pediatric asthma care on health care utilization, asthma outcomes, and QoL, including additional information about longer-lasting effects after cessation of eHealth care. Moreover, the holistic eHealth monitoring of physiological, environmental, and disease management data allows a comprehensive set of secondary analyses for insight into pediatric asthma. This study may therefore contribute to the existing knowledge of eHealth applications in secondary health care and to a better understanding of the value of home-monitoring diagnostics for pediatric asthma.

### Strengths and Limitations

This study is a follow-up of a quasi-experimental eHealth evaluation study [34]. Therefore, the involved HCPs of the hospital are experienced with using eHealth technology, and the care pathway is already aligned to deliver remote care in a safe and efficient way, which are commonly the most important barriers to successful eHealth evaluation trials [52]. Moreover, improvements based on prior eHealth trials within our hospital are integrated into this protocol to maximize its feasibility.

Another strength of the ALPACA study is the fact that it uses a set of home-monitoring devices chosen to be as unobtrusive as possible to minimize the daily living burden of the participating children and their parents. The current literature shows an increase in the development and evaluation of these unobtrusive sensing systems, enabling adherent long-term use for monitoring chronic disease course and management [53]. Additionally, this study uses a communication platform to provide children and their parents the opportunity to unobtrusively share a request for help or advice and integrate time- and event-based repeated home monitoring measurements or diary entries to obtain valid, real-life self-reports [42].

Objectified feedback to participants in eHealth care about inhalation technique (via videos) and therapy adherence (via smart inhaler data) could be a great addition to the current asthma care for children [54], as inhalation technique and therapy adherence appear to be poor but often remain “the elephant in the room” and stay unaddressed [55-58]. Automated synchronization of the mobile device and the inhaler add-ons provides a burden-free way to increase awareness of therapy adherence among children with asthma and their parents and provides HCPs with the necessary information to make informed medication adjustments [34,59].

Since a critical evaluation of financial cost-effectiveness is lacking in the literature for electronic health tools in pediatric asthma [60], the design of the ALPACA study is favored, as all direct hospital health care utilization is registered in combination with asthma outcomes. This gives us the opportunity to perform a thorough cost analysis of the care transformation to eHealth care. Moreover, the follow-up period of this study allows us to investigate whether the effect may endure when eHealth is ceased, providing interesting information on the possible long-term benefits of eHealth care.

A limiting factor in eHealth studies may be the timing of inclusion, as the asthma outcomes are subject to the episodic variation of asthma (viral infection, weather conditions, and pollen seasons) and COVID-19 regulations (lockdowns versus free contact) [2,61]. This effect is canceled to a great extent by the randomized design of this study. However, this study may be limited in the secondary pre-post evaluation of the different eHealth intervention elements. In addition, the worldwide generalizability of this study may be limited based on the eligibility criteria (children who live >80% of the time at the same home and have a stable WiFi connection).

Previous research has shown that the circadian rhythm influences the severity of asthma symptoms [62,63]. Symptoms tend to be worse during the night and gradually improve during the day. To avoid any bias of this circadian rhythm on the spirometry measurements, we will instruct all participants to perform the standard monitoring measurements at approximately equal times prior to dinner.

### Implications for Pediatric Asthma Health Care

The results of the ALPACA study may benefit future pediatric asthma health care in several ways. Researchers and the technology industry will be able to tease out relevant intervention elements that can act as digital markers for composing effective interventions, which can guide them in appropriate eHealth development [35]. Moreover, hypothesized health care utilization reductions provide positive incentives and increase the sense of urgency among stakeholders to take joint action toward scalable eHealth solutions [64]. Additionally, HCPs, health care institutions, and policy makers may use the ALPACA results to make informed decisions to steer toward a new, high-quality efficient pediatric asthma care pathway that goes beyond current traditional planned follow-up care and evolves toward proactive and targeted care, wherein the patient is actively involved via shared decision-making and self-management while being safeguarded by home-monitoring technology.

## Appendix

Multimedia Appendix 1 Outcome measures and statistical analysis of the secondary parameters.

## Abbreviations

ALPACA: Ambulatory Pediatric Asthma Care
C-ACT: Childhood Asthma Control Test
CE: Conformité Européenne
EU: European Union
GDPR: General Data Protection Regulation
HCP: health care professional
MST: Medisch Spectrum Twente
QoL: quality of life
SPIRIT: Standard Protocol Items: Recommendations for Interventional Trials
SpO2: oxygen saturation
