# Supplementary material for: Remote Patient Monitoring and Teleconsultation to Improve Health Outcomes and Reduce Health Care Utilization of Pediatric Asthma (ALPACA Study): Protocol for a Randomized Controlled Effectiveness Trial
Source: JMIR Res Protoc. 2023 Jul 3;12:e45585. doi: 10.2196/45585 (PMC10365621; doi:10.2196/45585)
Supplement: Multimedia Appendix 1 [file resprot_v12i1e45585_app1.docx]

*APPENDIX 1: Outcome measures and statistical analysis of the secondary parameters:*

**Asthma outcomes and quality of life:**

Besides the health care utilization and costs, the ALPACA study will investigate the effects of eHealth care on asthma outcomes and quality of life after 3 months of the eHealth intervention. One of the asthma outcomes is the asthma control score, assessed with the childhood asthma control test. The other asthma outcome is the lung function. The forced expiratory volume in 1 second (FEV1) is assessed with the home spirometer and is normalized according the Global Lung Initiative (GLI) reference set. Two questionnaire scores; the PAQLQ (total score and separated symptom, activity and emotional domain scores) and the EQ-5D-Y are used as quality of life outcome measures.

The statistical analysis of the asthma and quality of life outcomes is identical to the univariate analysis of the primary outcomes.

**Learning effects:**

Moreover, the effects on health care utilization, asthma outcomes and will be evaluated again after an additional 3 months of follow-up compared to the control group, to investigate the size of the so called “learning” effects after cessation of eHealth. The duration (the time to the first emergency visit/admission after the intervention phase) will be investigated using the Kaplan-Meier survival analysis.

**eHealth intervention elements:**

By sub analyses, the effects of single eHealth intervention elements (labelled as treatment changes, educational/self-management advises, medication feedback) on asthma outcomes (lung function and C-ACT score) and self-management score (PAM13), which will be analyzed using a pre-post paired t-test (or non-parametric Wilcoxon signed rank test).

***Therapy adherence and inhalation technique:***

The effect of medication reminders, feedback on adherence (smartinhaler data) and feedback inhalation technique (videos) on the adherence (% of prescribed medication), inhalation technique (1-number of errors made/total numbers of errors that can be made with specific device, which is judged by specialized asthma nurse) is analyzed by using a pre-post paired t-test (or non-parametric Wilcoxon signed rank test).

***Spirometry technique:***

The effect of spirometry feedback (given by the specialized HCP’s) on the spirometry technique (%of correctly executed measurements assessed by the quality control of flow-volume curve or video assessment) will be analyzed using a pre-post paired t-test (or non-parametric Wilcoxon signed rank test).

***eHealth supervised nebulizer therapy:***

The nebulizer will be used in case of an exacerbation, the numbers of use will be counted and each event will be classified as successful (in case nebulizer use help the patient through the exacerbation without an hospital visit) or unsuccessful (in case symptoms remain despite nebulizer use and a hospital visit is needed). )

**Correlation of home-monitoring parameters with asthma control:**

All home-monitoring parameters (Table 1) will be univariately and multivariately correlated to the asthma outcomes (lung function & asthma control (both binary: controlled versus uncontrolled and continuous: (C-ACT score)).

Table 1: Home-monitoring parameters

| Rescue medication usage |
| --- |
| Adherence to control medication |
| Sleep (duration, efficiency, latency, awakenings, bedtime, wake-up-time, sleep restlessness) |
| Nocturnal breathing rate |
| Nocturnal heart rate |
| Nocturnal heart rate variability |
| Nocturnal coughing (amount, timing) |
| Nocturnal wheezing (amount, timing) |
| Air quality (PM2.5, CO2, temperature, humidity etc) |
| Lung function (regular, during symptoms, after reliever medication, after sports) |
| Oxygen saturation (SpO2). |

*Univariately*

The differences across the asthma groups in the variables that did not have a normal distribution will be tested with the Kruskal-Wallis test followed by multiple comparisons of Games-Howell. The difference of normally distributed variables across the asthma groups will be tested with Analysis of Variance (ANOVA) followed by Tukey HSD test for the post-hoc comparisons of the three groups. P-values less than 0.05 are considered as significant.

*Missing data*

Prior to the multivariate analysis missing data will be handled using the multiple imputation regression method. Missing data patterns are analyzed for monotonicity. In case of monotonicity the monotone method is used; in case of random patterns the Markov Chain Monte Carlo method is used. Constraints will be added to the variables to prevent unrealistic imputations (e.g. negative lung function values). Five imputed datasets will be created and pooled according to the bar procedure (47).

*Multivariately*

Multivariate analysis will be performed using a binary logistic regression analysis with asthma control as dependent variable, with the controlled asthma group as reference group. All home monitoring parameters (see table 2) will be considered for inclusion in this final multivariate model.  Independent variables with a multi-collinearity of more than 0.8 will not be used both in the same model. The model was not adjusted for other potential predictors, such as age, gender, allergies etc. Stepwise forward likelihood ratio selection was used as enter method of variables with an entry probability of 0.10 and removal probability of 0.20. The model was optimized using the Nagelkerke pseudo R-squared, so that the model which explained the most of the variation (R^2^ closest to 1.0) was chosen. The resulting binary logistic regression was used to determine relevant diagnostic validity measures, such as sensitivity, specificity and positive and negative predictive value.

**Predict exacerbations:**

Machine learning based generative models will be used to detect and predict (behavioral modeling) exacerbations in time evolving data in a semi-supervised manner, by making use of learned knowledge and some expert knowledge. Parametric and non-parametric predictive machine learning models such as ordinary least squares (OLS), generalized linear models (GLM) or least square support vector machines (LSSVM) will be considered for the realization of the predictor. Deviations from normality will be mapped and a system will be developed to predict the exacerbation of asthma with the currently used home-monitoring parameters.

**The symptom perception:**

During this study the asthma symptom perception is quantified as the correspondence between the between self-reported dyspnea and home-measured lung function. Dyspnea is self-reported with a visual analogue scale (0-10) before each lung function measurement (FEV1 % pred). All the results of the home-monitoring measurements are plotted in the Perception Rainbow plot (part of current trial: <https://www.trialregister.nl/trial/9638>), figure 2. Corresponding areas in the Perception Rainbow were graded with a score ranging from 1 to 16.


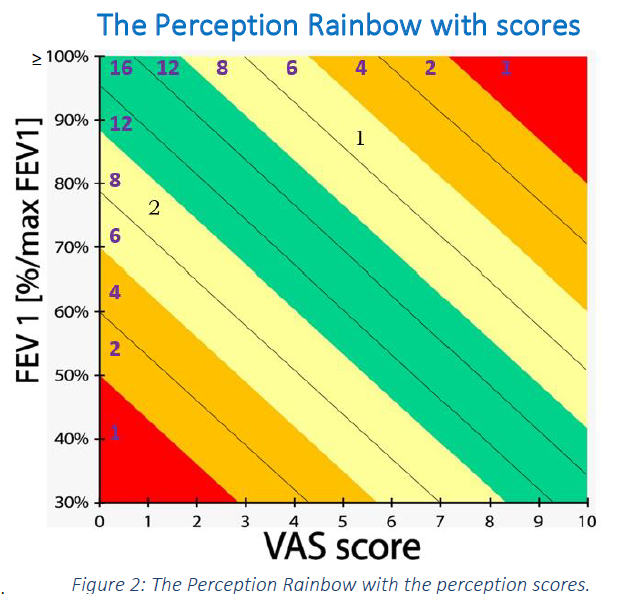


Figure 1: The perception rainbow with the perception scores. Two measurement examples (1 and 2) are plotted and correspond to a score of 6 and 8 respectively.

Moreover, the correlation between the VAS score and the lung function values (FEV1) between every measurement will be analyzed. Patient specific correlation coefficients will be inspected. If there is a large variation between subjects, univariate/multivariate analysis may be performed to analyze whether specific patient characteristics can predict the level of symptom perception of the children.

**Patient characteristics versus eHealth success:**

In order to identify the patient characteristics of children with successful eHealth care outcomes, a univariate and multivariate correlation analysis will be performed. The dependent variable is eHealth success which will be the continuous variable: “reduction in health care costs compared to historical data (%)” and another variable of success which will be investigated is the compliance to home-measurements. Independent variables (patient characteristics) that will be considered are listed in table 2.

Table 2: Patient characteristics.

| Age |
| --- |
| Gender |
| Weight |
| Length |
| BMI z-score: in children it is important to use the z-score. |
| Ethnicity |
| Medication step (1,2,3,4,5) according to GINA guidelines. |
| Inhalation allergy (y/n + which): Skin prick test or RAST blood test. |
| (asthma related) medical history: Sports / daytime symptoms /nighttime symptoms / family history / prematurity / smoking / |
| Baseline lung function (FEV1 (L)) |
| C-ACT score |
| Health literacy parents (Health Literacy Survey (HLS)) |
| Educational background parents. |
| Self-management (PAM-13 questionnaire) |
| Quality of care (CSQ-8) |
| Historical health care utilization |
| Medication adherence |

**User satisfaction & experience of eHealth care:**

Several questionnaires are used to investigate to effects eHealth has on the subjects in terms of self-management (Patient Activation Measure-13), health literacy (Health Literacy Survey). Moreover, quality of care (Client Satisfaction Questionnaire-8) is evaluated. The difference in questionnaire scores between the start and end of the intervention period (t_1_), will be compared with the control group using a two sample t-test (or non-parametric Mann-Whitney-U test).

***Feasibility & Acceptance:***

Technical feasibility was assessed by technology use, system usability, and technology acceptance. Technology use was determined continuously by the number of chat messages, time to respond, time spent using the Engage portal (minutes/week), and the adherence (%) of the spirometry data uploads (assuming 1 lung function measurement per week). System usability and technology acceptance will be assessed using the System Usability Scale and Technology Acceptance Model at the end of the eHealth program, and in addition, by means of a nondirective interview (with an average duration of 5 min) in which the children and parents are asked to provide their experiences of using the technology as part of the eHealth program. From the interview, an overview of the issues is made by grouping similar issues and those were converted into categorical codes (0=negative, 1=positive) to allow for statistical analyses. Moreover, the issues will be categorized as minor, serious, or critical based on the frequency and consequences and verified by the involved HCPs. All these outcome will be explained using descriptive statistics.
